# Calculi in Three of the Four Ureters in a Case of Bilateral Complete Duplication, Complicated by Pyonephrosis in One Obstructed Moiety—A Rare Case Report

**DOI:** 10.1155/criu/6380877

**Published:** 2026-04-16

**Authors:** Swanand E. Chaudhary, Ved Mahajan, Ritvij Patankar, Rohit Nimje

**Affiliations:** ^1^ Department of Surgery, NKP Salve Institute of Medical Sciences, Nagpur, India

**Keywords:** bilateral ureteral duplication, ureteric calculi, ureteroscopy

## Abstract

Duplex ureters, a urinary tract anomaly affecting 0.8% of the population, are typically asymptomatic. This case report describes an exceptionally rare presentation of bilateral complete ureteral duplication with calculi in three out of four ureters and pyonephrosis in one obstructed renal moiety. Although most cases of ureteral duplication are incidental findings, this report highlights a rare instance where bilateral complete duplication presented with symptomatic complications requiring intervention.

## 1. Introduction

Of all the congenital urological anomalies, renal duplication is among the most common observed pathologies, occurring in 1%–2% of the general population. Urinary tract anomalies occur commonly (3% of live births). Of the many malformations, duplex ureters occur in 0.8% of the population (i.e., 1 in 125), with a female‐to‐male ratio of 1.6 to 1 [1]. Duplex ureters may be unilateral or bilateral, incomplete, or complete.

Unilateral duplication is six times more common than bilateral [1]. Incomplete duplication (both ureters join to become one ureter before entering the bladder) is three times more common than complete duplication (both ureters open separately in the bladder) [1]. Bilateral complete duplication is rare, and the incidence is 1 in 500 (0.2%). It is found in 0.3% of excretory urograms [2].

Most ureteral duplications are asymptomatic and diagnosed incidentally. We report an extremely rare case of bilateral complete ureteral duplication with calculus in three of the four ureters with pyonephrosis in one obstructed moiety. Only a few case reports of bilateral complete ureteric duplication with ureteral calculi have been reported in the literature [3, 4].

## 2. Case Summary

A 53‐year‐old hypertensive, nondiabetic male presented with a history of vague upper abdominal pain, mild lower urinary tract symptoms (LUTS), and intermittent fever for 3 months. There was no other significant past history except an episode of lithuria 25 years ago. On examination, vital signs and abdominal findings were normal. Urine examination showed 50–60 pus cells, and *E. coli* was isolated on culture. Complete blood count (CBC) and kidney function tests (KFT) were normal.

**Figure 1:**
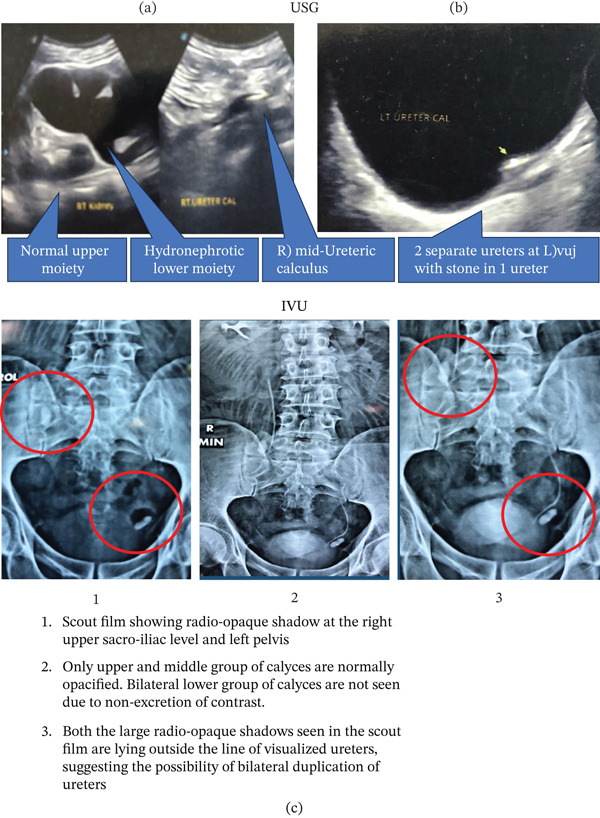
(a) The ultrasonography of the right kidney and mid‐ureter showing normal upper moiety and hydronephrotic lower moiety due to a large mid‐ureteric calculus. (b) The ultrasonography of the urinary bladder showing two ureteric openings at the left vesicoureteric junction and a calculus in one ureter. (c) The scout film showing radio‐opaque shadow at the right upper sacroiliac level and left pelvis. Only the upper and middle group of calyces are normally opacified. Bilateral lower group of calyces are not seen due to nonexcretion of contrast. Both the large radio‐opaque shadows seen in the scout film are lying outside the line of visualized ureters, suggesting the possibility of bilateral duplication of ureters.

Ultrasonography (USG) of the abdomen and pelvis revealed a duplex system of the right kidney. The upper moiety was normal, whereas the lower moiety was grossly hydronephrotic due to a large mid‐ureteric calculus (Figure 1a). The left kidney showed minimal hydronephrosis in the visualized moiety. No upper ureteric calculus was seen. USG of the bladder demonstrated two ureteric openings at left vesicoureteric junction and a large calculus in one of the ureters (Figure 1b).

Subsequently, intravenous urography was performed. The scout film showed a large radio‐opaque shadow at the right upper sacroiliac level and another in the left pelvis. No radio‐opaque shadow was observed in the left kidney or upper ureteral region. Only the upper and middle group of calyces were normally opacified. The bilateral lower groups of calyces were not visualized due to nonexcretion of contrast. Both radio‐opaque shadows seen on the scout film were located outside the line of the visualized ureters, suggesting the possibility of bilateral ureteral duplication (Figure 1c).

During hospital stay, the patient developed an episode of fever, dysuria, and abdominal pain associated with a marginally elevated total leucocyte count and serum creatinine. The patient was managed by escalating antibiotic therapy. His symptoms improved with normalization of the leucocyte count, but the serum creatinine stabilized at 1.45 mg/dL.

**Figure 2:**
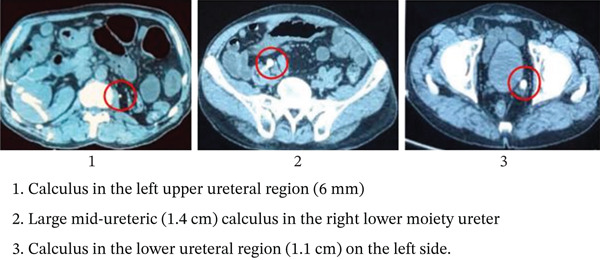
Noncontrast CT KUB. Calculus in the left upper ureteral region (6 mm). Large mid‐ureteric (1.4 cm) calculus in the right lower moiety ureter. Calculus in the lower ureteral region (1.1 cm) on the left side.

For further assessment, a plain CT scan of the abdomen was performed because the serum creatinine was elevated. It revealed a right duplex system, with a normal upper moiety and a grossly hydronephrotic lower moiety. The left kidney was small in size. A total of three calculi were identified: a large mid‐ureteric (1.4 cm) calculus in the right lower moiety ureter, and two calculi on the left side—one in the upper ureteral region (6 mm) and another in the lower ureteral region (1.1 cm) (Figure 2). As contrast was not used, the ureters were not delineated, and a conclusive diagnosis of duplicated ureters could not be made.

Before proceeding with surgery, DTPA renal scintigraphy was performed. The report was as follows:

• Right kidney
  o. Total: 76.8%
  o. Upper moiety: 76.8%
  o. Lower moiety: no uptake
• Left kidney
  o. Total: 23.2%
  o. Upper moiety: 96%
  o. Lower moiety: 4%

After this assessment and control of the urinary tract infection (UTI), we proceeded with the surgical treatment with a working diagnosis of bilateral complete duplicated ureters with three calculi.

Initial cystoscopy revealed mild benign prostatic hyperplasia (BPH), a normal bladder, and four orthotopic ureteric orifices at the trigone—two on each side of the interureteric ridge. This confirmed the diagnosis of bilateral complete duplicated ureters.

**Figure 3:**
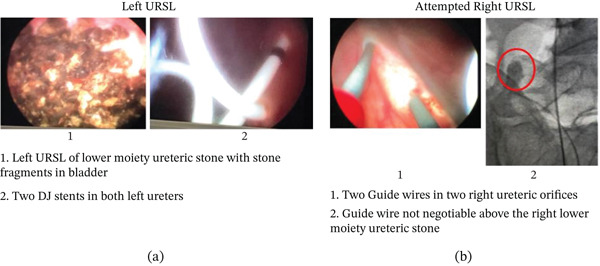
(a) Left URSL of lower moiety ureteric stone with stone fragments in bladder. Two DJ stents in both left ureters. (b) Attempted right URSL. Two guide wires in two right ureteric orifices. Guide wire not negotiable above the right lower moiety ureteric stone.

Both left ureteric calculi—one large calculus in the distal part of the lower moiety ureter and another small calculus in the proximal part of the upper moiety ureter—were completely cleared using semi‐rigid ureteroscopy (6–7.5 F) and pneumatic lithotripsy in a single session. Postprocedure, 6/26‐F DJ stents were placed in both left ureters (Figure 3a).

Semirigid ureteroscopy with 6–7.5 F ureteroscope was attempted in the right lower moiety ureter during the same session. However, the procedure was unsuccessful and had to be abandoned because the guide wire could not be negotiated above the stone due to tight impaction and severe kinking (Figure 3b).

**Figure 4:**
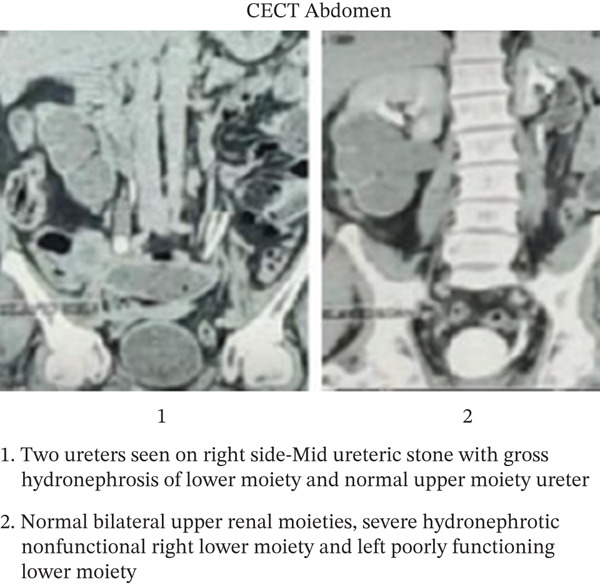
CECT abdomen. Two ureters seen on right side—Mid ureteric stone with gross hydronephrosis of lower moiety and normal upper moiety ureter. Normal bilateral upper renal moieties, severe hydronephrotic nonfunctional right lower moiety and left poorly functioning lower moiety.

After the first session of treatment, the serum creatinine decreased to normal levels. A contrast‐enhanced CT (CECT) scan of the abdomen was therefore performed, which reaffirmed the diagnosis of bilateral complete duplication of ureters (Figure 4).

In view of the CT and renal scintigraphy findings of a nonfunctioning hydronephrotic right lower moiety, heminephrectomy (HN) was advised, but the patient was not willing to undergo this procedure and preferred only stone clearance.

**Figure 5:**
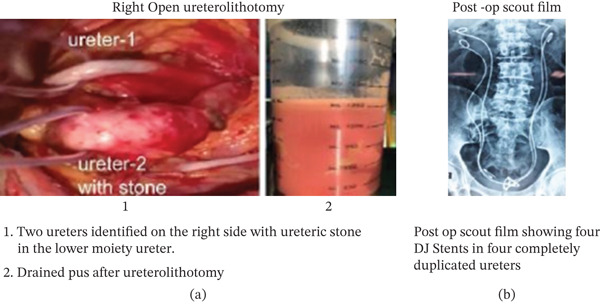
(a) Right open ureterolithotomy. Two ureters identified on the right side with ureteric stone in the lower moiety ureter. Drained pus after ureterolithotomy. (b) Post‐op scout film showing four DJ stents in four completely duplicated ureters.

The right mid‐ureteric stone was removed by open ureterolithotomy. Under regional anesthesia, the normal upper moiety ureter was initially stented with 6/26 DJ stent for proper identification and prevention of intraoperative injury. Retroperitoneal access was obtained through an oblique right iliac incision. Two ureters were identified. The ureteric calculus (1.4 cm) was removed by ureterotomy, and surprisingly, approximately 800 mL of pus was drained after stone removal. A 6/26‐F DJ stent was placed, and the ureterotomy was closed (Figure 5a).

The patient had an uneventful postoperative recovery and was discharged with four DJ stents in situ (Figure 5b).

After 8 weeks, the four DJ stents were removed, and the patient remains under follow‐up for future consideration of right lower HN.

## 3. Discussion

Bilateral complete ureteral duplication is a rare anomaly. In a series of 51,880 autopsies, ureteral duplication was observed in 0.66%, whereas in another autopsy series the ureteral duplication rate was 0.68% [5]. In a study of 800 cadaver kidneys procured for transplantation, only nine cases of duplicated ureters were identified, with an incidence rate of 1% [5]

The condition arises during the fourth to fifth week of gestation when two separate ureteric buds emanate from a single Wolffian duct. The urinary system develops from the intermediate mesoderm and ureteral duplication results when the ureteral bud bifurcates before it reaches the metanephric blastema. When the two ureteric buds originate from the mesonephric duct, it results in complete duplication, and when a single ureteric bud bifurcates, it results in incomplete duplication [6].

Kuang et al. [7] reported a rare case of caudal duplication syndrome. The patient was a male neonate with upper and lower urinary tract malformations including complete diphallia, a duplicated bladder, bifid scrotum, duplex kidneys (DKs), and extranumerary kidneys. The presence of DKs in this patient was one of the many congenital malformations.

Although renal duplication is relatively common, many cases remain underreported due to their asymptomatic nature. The subtle nature of the condition often makes diagnosis difficult. The anomaly may even remain unrecognized when one of the two renal moieties is small and dysplastic.

In our case, the left lower moiety was small and atrophic, and the diagnosis was not possible on USG or noncontrast CT (NCCT) of the abdomen. CECT of the abdomen, renal scintigraphy, and cystoscopy established the diagnosis on the left side.

Symptomatic presentation usually occurs due to associated conditions such as collecting system obstruction, urolithiasis, ureterocoele, ectopic ureter, vesicoureteral reflux (VUR), and UTI [8]. In children with ureteral duplication, the risk of renal infection increases approximately 20‐fold [5, 9].

The association of bilateral complete ureteral duplication with urolithiasis is even rarer [10]. The unique anatomical configuration of duplicated kidney and ureteral malformation predisposes it to urinary stasis, thereby increasing the probability of urinary stones formation [2, 11].

Only a few reports of bilateral complete ureteral duplication with urolithiasis exist in the literature. Aiken et al. reported a patient with bilateral complete ureteral duplication and calculi obstructing both limbs of the left duplicated ureter [3]. Ahmed reported a case of bilateral complete ureteral duplication with obstructing calculi in all four ureteric limbs [4].

In patients with DK and ureteral systems affected by calculi, efforts should be directed to know the location, size, number of calculi along with the assessment of ureteral malformation, location of ureteric orifices and the functional status of renal moieties. Imaging modalities like intravenous pyelography (IVP), CT urography, MR urography, cystoscopy, and retrograde pyelography (RGP), in various combinations, are required for a thorough assessment.

Anatomical variations of the ureter are of great importance to surgeons, gynecologists, and urologists because they increase the risk of iatrogenic ureteral injury. Ureteral injuries are serious complications of pelvic operations and may increase morbidity and even cause mortality. The American Urologic Association (AUA) guidelines for urolithiasis recommend obtaining additional imaging in cases with complex anatomy [12].

However, Chertack et al. reported that preoperative knowledge of ureteral duplication has no significant effect on the safety or efficacy of ureteroscopy [13].

In a retrospective study on ureteroscopic lithotripsy (URSL) in patients with ureteral duplication, Luo et al. found that the diagnostic accuracy of CT for such anomalies was only 61.9% [14]. This suggests that the diagnostic accuracy of CT for such anomalies needs improvement. Three‐dimensional reconstruction may further enhance diagnostic accuracy.

In our case, CT urography combined with cystoscopy established the diagnosis of bilateral complete ureteral duplication with urolithiasis.

Our patient had four complete ureters, with orthotopic normal ureteric orifices and calculi involving three ureters. The bilateral lower renal moieties were significantly affected by gross hydronephrosis, atrophy, poor function, and UTI.

We successfully managed both left‐sided ureteric calculi using semirigid ureteroscopy (6–7.5 F) and pneumatic lithotripsy in a single session without prior stenting.

Aiken et al. reported the need for prior ureteral stenting due to difficulty in ureteroscopic access. Elective URSL was done 4 months later [3]. However, Ahmed successfully managed all four ureteric calculi in bilateral complete duplication using a semirigid 6–7.5‐F ureteroscope with Holmium laser. They managed the case in a single session without prior stenting [4].

The large right mid‐ureteric calculus in our patient required open surgery. Although a minimally invasive laparoscopic or robotic approach could have been considered, limitations in resources and expertise prevented their use.

The drainage of approximately 800 mL of pus after right ureterolithotomy was a surprise intraoperative finding, as there was no clinical or imaging evidence of pyonephrosis preoperatively. This could be due to the masking of symptoms by the administration of higher antibiotics in the initial phase of treatment. If clinical or imaging evidence of pyonephrosis had been present, preoperative urinary diversion followed by staged definitive procedure would have been necessary.

In general, the management strategy of bilateral complete ureteral duplication depends on the coexisting issues like VUR, ureterocoele, ectopic opening of ureter, UTI, and the functional status of the four renal moieties. A thorough anatomical and functional assessment is required for optimal treatment plan.

The treatment approach must consider the unique anatomy of four ureters and four renal moieties and their functional status. The choice of surgical procedure is therefore highly individualized and tailored to the underlying pathology. The primary goals are to alleviate the clinical symptoms, prevent progressive renal damage, and restore functionality to the upper and lower urinary tracts.

Depending on the clinical presentation, which may involve obstructive or reflux pathologies, a range of minimally invasive endoscopic, laparoscopic, or open surgical approaches may be employed for extirpative or reconstructive purposes or a combination of both.

Although there is no universally established classification system for pathologies associated with DKs, the patients can broadly be grouped according to the following characteristics:

• Unilateral or bilateral DKs
• Hydroureteronephrosis of an upper and/or lower renal unit
• Presence of VUR
• Presence of a ureterocoele, ectopic ureter, or ureteropelvic junction obstruction (UPJO)

Surgical treatments could be grouped as follows:

• Unilateral or bilateral surgery for pathologies in unilateral or bilateral DKs
• Upper tract, lower tract, or combined approaches
• Endoscopic treatment (either alone or preceding open surgery)
• A single or staged procedure
• Additional surgery on the contralateral non‐DK

In a retrospective study of 176 patients, Ujkic et al. [15] reported the following distribution of surgical treatments for DK and associated pathologies.

An upper tract approach was used in 20 patients, including nephrectomy (*n* = 2), unilateral HN (*n* = 13), unilateral pyelo‐pyeloplasty (*n* = 1), and unilateral uretero‐pyeloplasty (*n* = 4).

A lower tract approach was used in 98 patients, including unilateral ureteroneocystostomy (*n* = 70), bilateral ureteroneocystostomy (*n* = 16), bilateral distal uretero‐ureterostomy (*n* = 1, staged procedure), unilateral endoscopic bulking (*n* = 8), and unilateral endoscopic ureterocoele decompression (*n* = 3).

A combined approach was used in 58 patients, including a unilateral upper tract procedure and ureteral stump excision (USE, *n* = 22), a unilateral upper tract procedure and ureteroneocystostomy (*n* = 30), and bilateral HN and ureteroneocystostomy (*n* = 6). Fourteen of these combined surgeries were performed as staged procedures.

Both obstructive and refluxing pathologies were treated using an upper tract, lower tract, or endoscopic approach to perform endoscopic, extirpative, or reconstructive surgery or a combination thereof.

Because of the wide range of associated pathologies, it is difficult to establish a definitive algorithm for the management of DKs.

Guoqing Liu et al. [10] reported a case of multiple renal and ureteric calculi in a case of bilateral DKs. The patient had bilateral DKs, right incomplete and left complete ureteral duplication, with bilateral large renal calculi and left ureteral calculus. The patient was managed in three sessions:

1. Left URSL of the ureteric stone with DJ stenting of the second ureter.
2. Left percutaneous nephrolithotomy (PCNL).
3. Right dual‐channel PCNL.

They recommended detailed preoperative evaluation to determine stone location, stone size, and location of ureteral opening. They also recommend that treatment priority should be given to the kidney affected by complete duplication, ureteral stones, and the side affected by more severe hydronephrosis. They also recommended preoperative DJ stenting followed by a staged elective procedure.

In our case as well, complete stone clearance required two treatment sessions.

Considering the rarity of duplicated systems with urolithiasis and their varied presentation, a uniform algorithm of treatment is difficult to formulate.

In our opinion, following management principles for stone disease in DKs should be considered:

1. Thorough preoperative evaluation by various modalities like CT urography/IVP/MRI and cystoscopy is mandatory in DKs.
2. For ureteroscopic treatment, preoperative placement of a DJ stent for a few days may improve the success rate and reduce intraoperative complications.
3. Clinical or imaging evidence of pyonephrosis necessitates preoperative urinary diversion followed by a staged definitive procedure.
4. When both ureteric and renal calculi in DKs are present, stone clearance is safer when performed in more than one session.
5. The issues unique to bilateral complete duplicated ureters are the relatively narrow ureteric orifices in a common ureteral sheath. This potentially increases the chance of injury in an endourological procedure. It is preferable to use the smallest size of ureteroscope and ureteral access sheath if flexible ureteroscopy is contemplated.
6. Flexible ureteroscope with laser lithotripsy should be preferred over pneumatic lithotripsy to reduce the chances of retropulsion in the kidney.
7. With advances in flexible ureteroscopes and lasers, most calculi in anomalous kidneys and ureters can now be successfully treated.
8. Placement of the ureteral access sheath should be performed cautiously in the presence of ureteral anomalies. In an incomplete duplication, the ureteral access sheath should be ideally positioned just below the bifurcation.
9. Management principles of treatment of renal calculi in DKs are the same as for normal kidneys, including ESWL, PCNL, and RIRS.
10. Minimally invasive laparoscopic or robotic approaches may be used for large ureteric stones that are not amenable to ureteroscopic management.

## 4. Conclusion

To the best of our knowledge, this is the first reported case of bilateral complete duplicated ureters with urolithiasis involving three of the four ureters, complicated by pyonephrosis in one moiety and managed with a combination of endourological and open surgical procedures.

Clinical presentation and several factors determine the management strategy. These include the morphology and function of the duplex system, associated infection, reflux, location, size, and number of calculi, presence of ureterocoele, and ectopic or orthotopic ureteric orifices.

This case highlights the critical importance of meticulous diagnostic assessment and individualized endourological and open surgical treatment.

No written consent has been obtained from the patients as there is no patient identifiable data included in this case report/series.

## Funding

No funding was received for this manuscript.

## Conflicts of Interest

The authors declare no conflicts of interest.

## Data Availability

The data that support the findings of this study are available from the corresponding author upon reasonable request.
